# Development of mWellcare: an mHealth intervention for integrated management of hypertension and diabetes in low-resource settings

**DOI:** 10.1080/16549716.2018.1517930

**Published:** 2018-09-26

**Authors:** Devraj Jindal, Priti Gupta, Dilip Jha, Vamadevan S. Ajay, Shifalika Goenka, Pramod Jacob, Kriti Mehrotra, Pablo Perel, Jonathan Nyong, Ambuj Roy, Nikhil Tandon, Dorairaj Prabhakaran, Vikram Patel

**Affiliations:** a Clinical Trials, Centre for Chronic Disease Control (CCDC), New Delhi, India; b Centre for Control of Chronic Conditions, Public Health Foundation of India, New Delhi, India; c Global Services, Dimagi Software Innovations Pvt. Ltd, New Delhi, India; d Centre for Global Non Communicable Diseases, London School of Hygiene & Tropical Medicine, London, United Kingdom; e Centre for Control of Chronic Conditions, All India Institute of Medical Science, New Delhi, New Delhi, India; f Department of Global Health and Social Medicine, Harvard Medical School, Boston, MA, USA

**Keywords:** Clinical decision support system, complex intervention, evidence-based management, longitudinal patient monitoring, primary care, noncommunicable diseases

## Abstract

**Background**: Cardiovascular diseases and diabetes are among the leading causes of premature adult deaths in India. Innovative approaches such as clinical decision support (CDS) software could play a major role in improving the quality of hypertension/diabetes care in primary care settings.

**Objective**: To describe the steps and processes in the development of mWellcare, a complex intervention based on mobile health (mHealth) technology.

**Methods**: The Medical Research Council framework was used to develop mWellcare in four steps: (1) identify gaps in usual care through literature review and health facility assessments; (2) identify the components of the intervention through discussions and consultations with experts; (3) develop intervention (clinical algorithms and mHealth system); and (4) evaluate acceptability and feasibility through pilot testing in five community health centers.

**Results**: Lack of evidence-based, integrated, and systematic management of chronic conditions were major gaps identified. Experts in information technology, clinical fields, and public health professionals identified intervention components to address these gaps. Thereafter, clinical algorithm contextualized to primary care settings were prepared and the mWellcare intervention was developed. During the 2-month pilot, 631 patients diagnosed with hypertension and/or diabetes were registered, with a follow-up rate of 36.2%. The major barrier was resistance to follow mWellcare recommended patient workflow, and to overcome it, we emphasized onsite training and orientation program to cover all health care team member in each CHC.

**Conclusion**: A pilot-tested mWellcare intervention is an mHealth system with important components, i.e. integrated management of chronic conditions, evidence-based CDS, longitudinal health data and automated short-messaging service to reinforce compliance to drug intake and follow-up visit, which will be used by nurses at primary health care settings in India. The effectiveness and cost-effectiveness of the intervention will be tested through a cluster randomized trial (trial registration number NCT02480062).

## Background

Noncommunicable diseases (NCDs) account for 71% of global deaths, an increase of 14% from 2005 to 2015[1]. Three-fourths of these NCD deaths occur in low and middle-income countries [2]. In India, the probability of dying prematurely from the four main NCDs is 26.2%; among the four main NCDs, cardiovascular diseases (CVD) and diabetes are leading causes [2]. The population level prevalence of hypertension and diabetes in adults in India is high, with estimates of 23% [2] and 8.7% [3] respectively. However, studies have shown that management of hypertension, cardiovascular disease, and diabetes at primary care level is suboptimal [4–7], and only half of the patients achieve their target blood pressure and blood sugar level, which increases their risk for CVD events [4,5]. Some of the challenges in treating and managing chronic conditions are suboptimal adherence to management guidelines by the physicians [5,8], lack of regular patient monitoring and undiagnosed comorbidities such as anxiety, depression, and alcohol use disorders which can contribute to failure to follow treatment plans [9–11]. Mobile health (mHealth) technologies are being advocated to strengthen the health system, and systematic reviews have shown that an mHealth-based Clinical Decision Support System (CDSS) improves preventive care and the physician’s clinical decision quality in hypertension and diabetes management [8] although the evidence on their effects on cardiovascular outcomes is limited [12].

Considering the potential of mobile technology to address some of the challenges in chronic disease management, we sought to develop a tablet-computer-enabled mHealth intervention (called mWellcare) with the goal of integrated management of hypertension, diabetes, and other comorbid conditions at the primary health care level in India. This intervention is intended to be used by general health care providers (nurses/physicians) in primary care settings. The aim of this paper is to describe the steps and processes in the development and design of the mWellcare intervention.

## Methods

### Study setting

Frontline public health facilities in rural India consist of Primary Health Centers (population coverage approximately 20,000–30,000) and community health centers (CHCs) (population coverage approximately 80,000 – 120,000). Together these facilities cater to the majority of the primary care needs in rural India [13]. Under the National Program for prevention and control of Cancer, Diabetes, Cardiovascular disease, and Stroke (NPCDCS), NCD clinics are established at CHCs for screening, diagnosis, and management of common NCDs [14]. We based our study in CHCs in two states of India, Haryana in the north and Karnataka in the south. This study was approved by the institutional Ethics Committee at Public Health Foundation of India (PHFI) Reference No. TRC-IEC-107/11 and London School of Hygiene and Tropical Medicine (LSHTM) Reference No. 10,423.

### Research framework

In order to describe the steps and processes in the development and design of the mWellcare intervention, we used the framework for the development of ‘complex’ interventions proposed by the Medical Research Council (MRC), UK [15]. Based on this framework, we followed the following steps: (1) identifying gaps in usual care, (2) identifying the components of the intervention, (3) developing intervention components, and (4) evaluating acceptability and feasibility through pilot testing. We describe each step in the sequence, demonstrating how findings from each step were carried forward to subsequent steps.

### Step 1: identifying gaps in usual care

We identified gaps in usual care through our previous mHealth project experience [16–19], a literature review, and situation analysis of a sample of health facilities. We searched the PubMed database for peer-reviewed journal articles assessing the gaps in the management of hypertension and diabetes in India and other low- and middle-income countries published from January 2000 to December 2014. All possible combinations of the following keywords were used: hypertension, diabetes mellitus, clinical practice patterns, quality of health care, awareness, control, treatment, management, prescription pattern, adherence/compliance to medications, access to care, barriers, delivery of health care, health care disparities, lifestyle, follow-up, medication, patient-centered care, physician–patient relations, safety, primary, secondary, and tertiary care and India. We identified 1421 articles initially, of which 17 were relevant.

We conducted facility assessment at eight CHCs, four each in Haryana and Karnataka, to assess existing services, manpower, laboratory facilities, electricity, infrastructure, information systems, and drug supply. We also conducted semistructured interviews with physicians (*n* = 8), nurses (*n* = 6), other health functionaries [*n* = 13 (counselor (3) + Pharmacist (4) + Lab technician (6)] at CHCs to assess the existing patient-management practices, the extent of use of evidence-based guidelines for management of hypertension and diabetes, and the barriers and measures to improve the compliance to evidence-based guidelines by the healthcare team.

### Step 2: identifying the components of the intervention

We conducted consultation workshops (1) and meetings (3) to discuss the gaps (identified in above steps) with experts in information technology, clinical fields (endocrinologist, cardiologist, and psychiatrist), public health professionals, and government officials working in the NCD program. The number of participants in these meetings was from 10 to 25.

### Step 3: development of intervention

The mWellcare intervention was developed into two stages: (a) development of the clinical algorithms and (b) development of the mHealth application.

### Development of the clinical algorithms

The aim of this stage was to develop evidence-based clinical algorithms for the management of hypertension and diabetes and comorbid conditions (depression, alcohol use) for use at the primary health care level in India. National and international experts in cardiology, endocrinology, and mental health reviewed the latest national and international guidelines and selected those that were considered most suitable for the context: the American and International Society of Hypertension guidelines 2014 [20]; European Society of Hypertension 2013 [21]; WHO Guideline for Assessment and Management of Overall Cardiovascular Risk 2007 [22]; NICE guidelines [23]; NPCDCS medical officer manual [24]; and the mhGAP Intervention Guide for Mental, Neurological and Substance Use Disorders in Non-Specialized Health Settings 2010 [25]. These guidelines were adapted based on the available drugs and investigation facilities at the CHCs located in Haryana and Karnataka.

### Development of the application

After developing clinical algorithms, the team prepared the software specification document that included the detailed itemization of all questions and logic in the application. Based on the spec document, a prototype tablet-computer based application was developed by Dimagi (software development agency for mWellcare) on their CommCare platform. CommCare is an open-source mHealth platform for frontline service delivery, particularly in rural settings. It enables the execution of evidence-based health logic through its mobile platform, is scalable for large populations and will continue to evolve so as to reduce long-term maintenance and upgradation costs [26,27]. After internal testing of the application for the accuracy of content and clinical logic, multiple rounds of quality assurance (QA) testing were carried out by an external quality assurance agency (ValueLabs, Hyderabad, India) for user interface elements, Decision Support Recommendation (DSR) and accuracy of patients’ longitudinal data. After extensive iterative testing and refinement, the version for piloting in the real-world was finalized. In addition, automated short-messaging services (SMS) for self-management and follow-up to be sent to patients from the central server were developed and tested. Meetings between experts from different disciplines (medical, health communication, software, and telecom) were conducted to develop the content of the SMS. In these meetings, optimal content of the SMS in terms of language, type (text, picture, voice), optimal frequency of the SMS reminders, type of SMS reminders (drug intake and follow-up reminders), the recipient (patient/family member/friend) and alternative methods of reminders for patients such as voice messages and telephonic reminder were discussed, and SMS reminders were finalized.

### Step 4: pilot testing

The mWellcare intervention was piloted at five CHCs (two in Haryana’s Mewat district and three in Karnataka, two in Kolar district and one in Shimoga district) during April to July 2015 on newly diagnosed and existing patients with hypertension and/or diabetes. The objectives of pilot testing were: (1) to assess the acceptability, feasibility, and user-friendliness of the intervention, (2) to identify the barriers and challenges in intervention delivery and strategies to address them, and (3) to identify indicators for monitoring intervention delivery.

The intervention was rolled out in three stages:

Stage 1: Setting up the intervention
  This stage included obtaining information regarding nurses to create their user profiles in the application. Samsung tablets along with SIM cards, printers, weighing machines, stadiometers, BP apparatus and glucometers and strips for each CHC were procured. The mWellcare application was uploaded on the Samsung tablet.
Stage 2: Training of physicians and nurses
  We conducted 5-day training (3-day classroom and 2 days in-field) for nurses. The training was conducted in April and May 2015. In addition, a 1-day onsite training of physicians was conducted in each CHC.
Stage 3: Observation and support
  During the initial 3 days of mWellcare intervention delivery by the nurses in the NCD clinic, onsite observation and support were provided by the research and Dimagi teams.

This was followed by a 2-month period of monitoring and observation. The evaluation was completed by interviewing physicians (*n* = 5) and nurses (*n* = 5), obtaining field notes from the weekly observation visit by the research and Dimagi teams, and analyzing mobile user data from the central server. During this period, we monitored the process indicators such as number of new patients registered, follow-up rate, proper assessment (review of DSR by the doctor) and medication adherence.

### Trial registration

The mWellcare trial is registered with Clinicaltrial.gov (Registration number NCT02480062) and Clinical Trial Registry of India (Registration number CTRI/2016/02/006641).

## Results

### Gaps in usual care

Based on the literature review and facility assessment, we identified the following gaps:

1. Inadequate human capacity and resource: While all the CHCs had general duty medical officers, there were no dedicated doctors for NCD care. Six CHCs had NCD nurses while NCD counselors were available only in the four CHCs of Karnataka. None of the NCD nurses had received training to perform their duties at the NCD clinic. Physicians at CHCs reported limited access to evidence-based guidelines for the management of hypertension and diabetes. They also reported limited training and knowledge update regarding management of diabetes and hypertension.
2. Lack of systematic patient assessment and long-term management of chronic conditions: The health facility assessment showed that there is a lack of an organized system for patient screening, registration, record keeping, long-term follow-ups, monitoring of process indicators, and QA at NCD clinics.
3. Lack of integrated management of chronic conditions: Screening of common mental disorders and substance abuse is very rare in primary care, even though alcohol use disorder and common mental disorders like depression are associated with CVD risk factors and can contribute to failure to adhere to management plan among people with hypertension and diabetes [28].
4. Limited lifestyle advice: Physicians reported insufficient time to provide lifestyle advice and nurses had limited training for providing the same. Studies have shown insufficient diet control in diabetes, insufficient physician time to motivate and educate patients, reluctance or lack of motivation of patient for drug adherence and lifestyle changes as some of the reasons for suboptimal control of hypertension and diabetes [28–30].
5. Limited drug supply and diagnostics for hypertension and diabetes management: Only two classes of drugs for hypertension (beta blockers and calcium channel blockers) and diabetes (metformin and sulphonylureas) were available at the CHCs. Supply of other classes of drugs was irregular. While a sphygmomanometer and glucometer were available at all the CHCs, the supply of glucose test strips was inadequate. Other studies from India and other low- and middle-income countries have also shown similar deficiencies in drugs supply and equipment in primary care settings [31,32].

### Components of intervention

The consultation workshops and meetings led to the consensus on adopting an integrated approach to management of chronic condition rather than a disease-centric approach for patients with hypertension and diabetes in primary care settings using an mHealth application. Based on the consultation, components of the mHealth intervention and the gap it addressed were elaborated and synthesized (Table 1).

**Table 1.** Components of mWellcare intervention and the gaps in the usual care they address.

Component	Description	Gap addressed
Integrated management of chronic conditions	Screening for CVD risk, depression, and alcohol dependence using the CVD risk score, Patient Health Questionnaire (PHQ9) [33] and Alcohol Use Disorder Identification Test (AUDIT) questionnaire [34].	Lack of integrated management of chronic conditions, addressing common multiple morbidities
Evidence-based decision support to physicians and other health workers	Decision-support system to provide automated guideline-recommended treatment plans, referral to specialist, and lifestyle intervention tailored to individual patients	Inadequate human resource and capacity
Longitudinal health data	All patients diagnosed with hypertension and diabetes are registered and their clinical parameters recorded at every follow-up visit to provide a longitudinal trend/summary of the clinical parameters for decision-making	Lack of systematic patient assessment and long-term management of the chronic condition
Adherence support	Automated Short-Messaging Service (SMS) from a central server to reinforce compliance to drug intake and follow-up visit	Low adherence to long-term care
Lifestyle advice	The nurse will provide lifestyle advice to the patients based on relevant prompts provided by the application. All patients will be provided a brochure in the local language on lifestyle changes.	Limited patient lifestyle changes

### Intervention and its plan of delivery

**Figure 1.**
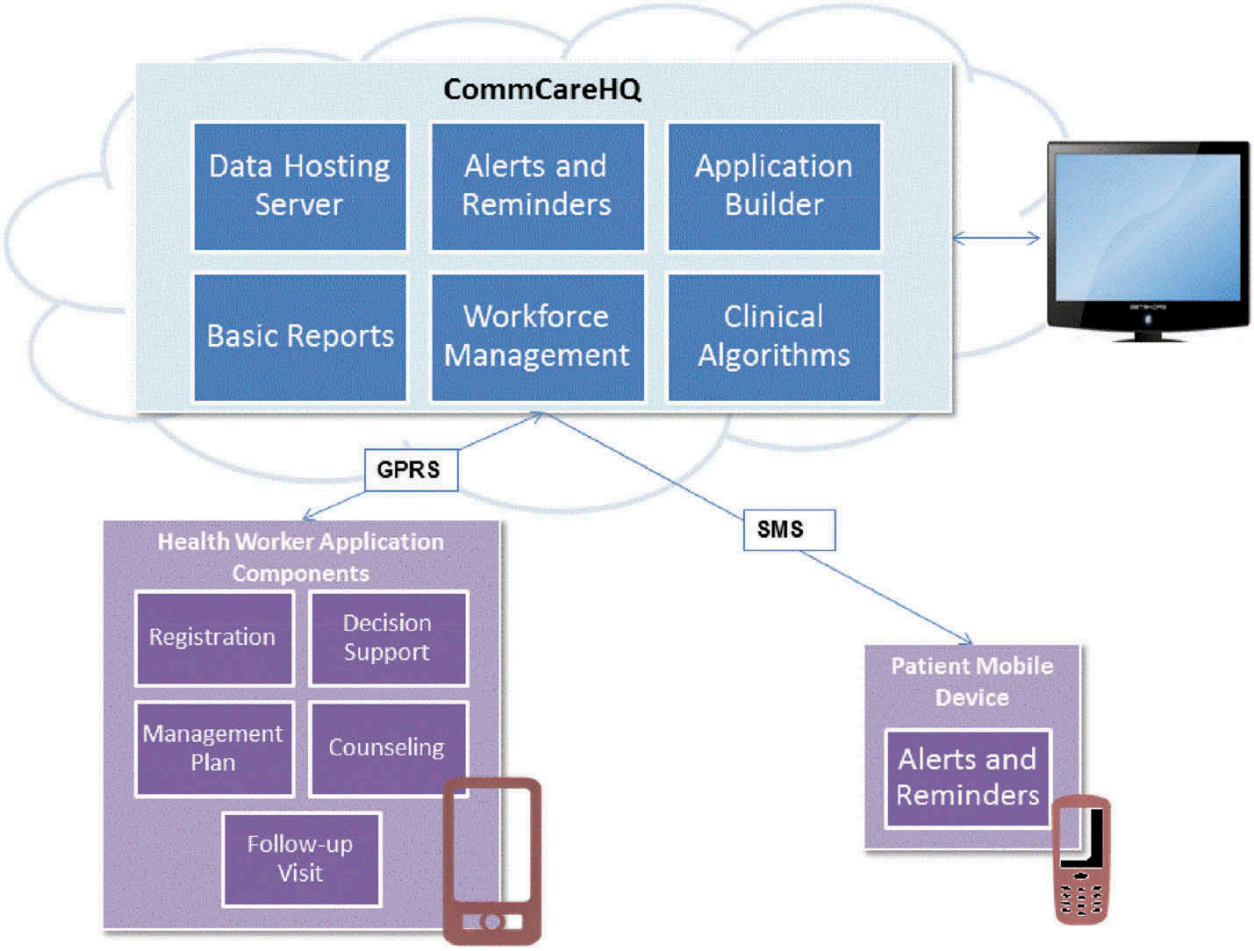
Architecture of mWellcare.

The resulting mWellcare intervention is a mobile health system consisting of a mobile component and a server component. The mobile application is customized for use by the primary care-based NCD nurse and doctor for maintaining longitudinal health records, decision support, patient case management, and generating a checklist for lifestyle advice. The NPCDCS in India has made provision for the NCD Nurse at the outpatient clinics of CHCs. The intervention aims to use the NCD Nurse for patient assessment and long-term follow-up using the mWellcare system thereby shifting/sharing some of the patient management tasks from doctors. During the time when the gaps in usual care were identified, doctors stated that they have a high OPD patient load which could pose operational challenges in using technology by themselves, but they showed positive interest in task shifting and having access to guideline-based decision support. The web interface of the system generates monitoring reports, user/domain management, and data viewing and extraction. A secondary user of the system is the patient. Consenting patients may receive customized messaging SMS messages on their mobile devices. Figure 1 demonstrates the architecture of the mWellcare intervention in terms of its functional components.

### Plan for delivery of the intervention

**Figure 2.**
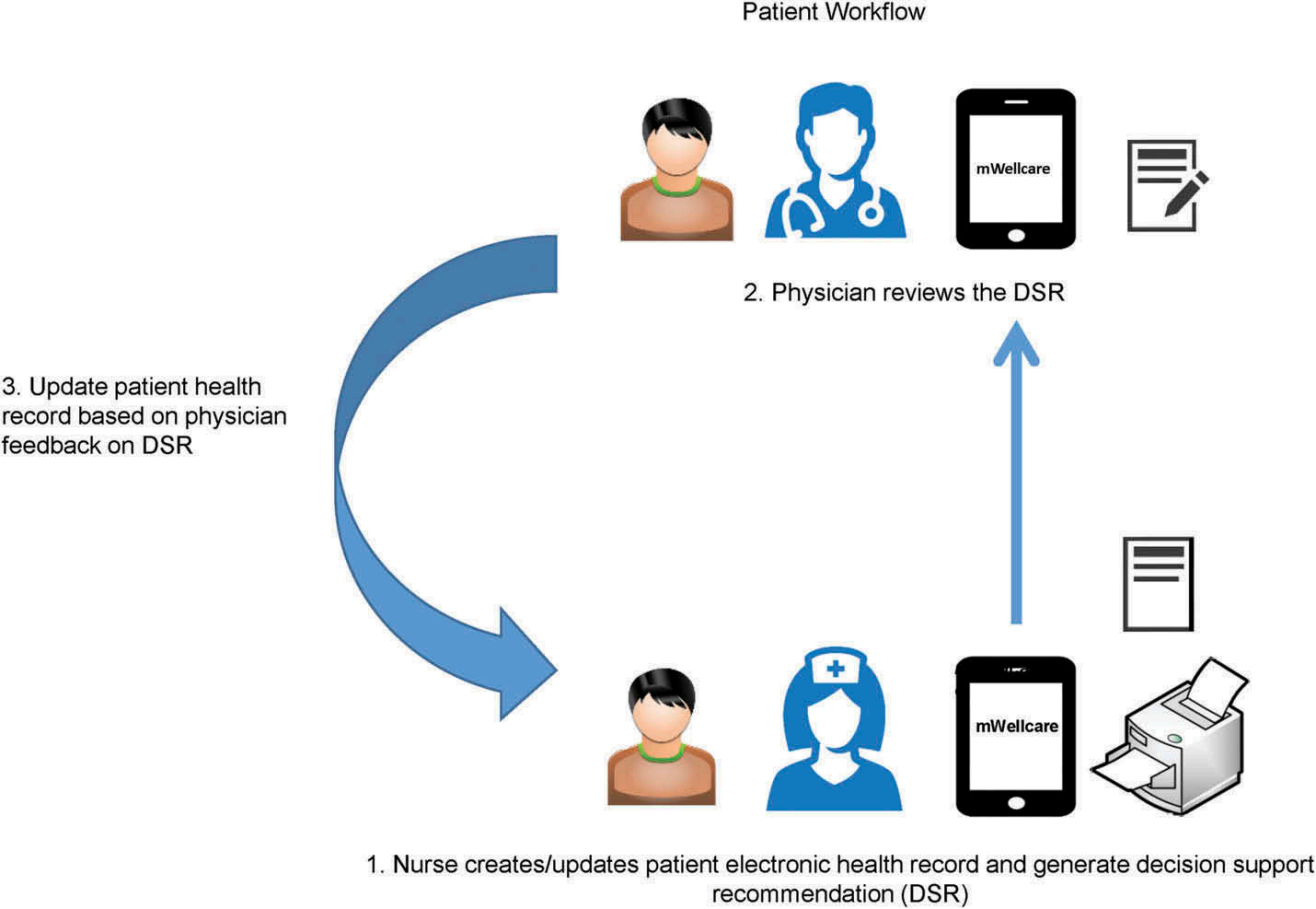
mWellcare workflow.

The application would be loaded on Samsung tablets (Tab-A, Android OS). NCD nurses were expected to log in to the application and register patients diagnosed with hypertension and/or diabetes by the physician. The NCD nurses would use the mWellcare system to conduct the initial patient evaluation and generate a printout of DSR. The patient would consult the physician with the DSR printout after initial evaluation by the nurse. After the physician’s feedback on the DSR, the patient would return to the NCD nurse, to update the physician’s final decision on DSR in the mWellcare application and receive lifestyle advice (Figure 2).

We anticipated resistance from the nurses, physicians, and health administrators to use mWellcare, as it involved significant changes in their workflow. We thus incorporated a number of change-management strategies including training and orientation of CHC healthcare team on the application; explaining the benefits of using mWellcare intervention for effective management of NCDs; inviting health care team feedback for the refinement of intervention; regular monitoring of the delivery of intervention through both remote tracking of application data and in-person visits; and involvement of higher level health officials in bringing about changes in the existing health system for effective delivery of intervention.

#### Pilot testing results

A total of 631 patients diagnosed with hypertension and/or diabetes were registered during pilot testing. Just over half were female (53%), the mean age was 59.7 years (58.8, 60.7), 65.6% of patients had hypertension, 14.7% had diabetes and 19.7% had both hypertension and diabetes. Physician agreement with hypertension and diabetes DSRs were 61% and 70%, respectively. The most common reason for disagreement was the unavailability of drugs recommended by the DSR. During the pilot, we observed that mWellcare workflow was not followed properly, because of a lack of communication between pharmacist, registration desk, nurses, and doctors regarding mWellcare intervention, resulting in a low follow-up rate of patients at NCD clinic. Out of 351 patients scheduled for the follow-up visit (as per clinical parameters), only 127 (36.2%) patients came back during the pilot testing period. Patients who came back for follow-up were asked about medication adherence, and we found that 98% of the patients with hypertension were taking antihypertensive medicines, and 61% of the patients with diabetes were taking antidiabetic medication. In patients with hypertension who came back for follow-up (119), the mean SBP and DBP at baseline was 150.4 and 87.2 and at follow-up was 145.5 and 86 respectively. Owing to the unavailability of glucometer strips at CHCs during the pilot period, no test was done for blood sugars during follow-up. We also observed that in 83% of cases, doctors provided their feedback on the DSR.

Nurses reported that the mWellcare intervention was helpful in performing their duties (for example, searching for patients, structured assessments) and improved their knowledge, skills, and ability to manage their workload. Physicians reported that the DSR printout was useful as it has reduced time spent and improved quality of patient assessment. Illustrative quotes are appended below.

‘Yes it is helpful because we treat patients in a different way now, so patients feel that staff are more responsible and working hard’ (Nurse)

‘My working skills have improved because previously I never used to work like this. mWellcare has helped me to learn new things’ (Nurse)

‘It has improved the quality of patient assessment, as it provides the patient summary at a glance in the DSR print out, but the unavailability of the drug is the major challenge in following the recommendations’ (Physician)

‘DSR output is cluttered and the name of the medicines is mentioned as the class name so we find it difficult in thinking of the generic name. DSR output should be made more readable, and the generic name of the medicine should be mentioned instead of the class name’ (Physician)

‘Patient assessment through this is time-consuming; sometimes patients get irritated and we have long queues in OPD’ (Nurse)

However, a number of barriers were observed and potential solutions were elaborated, some of which were tested during the piloting, as summarized in Table 2.

**Table 2.** Barriers encountered in the piloting of the mWellcare intervention and solutions.

Barrier	Solutions
**Application-related**	
Duplicate registration of patients	Added fuzzy search (by name, father’s name, phone number, etc.) to search for existing registered patient before opening new registration form
Inappropriate nomenclature of forms	Names of the forms were revised and replaced for the better understanding of the health care team (for example, ‘Confirmation of Treatment Plan’ form modified to ‘Record Prescription’ form)
Difficulty in administering the PHQ9 and AUDIT questionnaires in English	Local languages (Hindi and Kannada) versions of the questionnaires incorporated
DSR output and formatting issues, e.g. being lengthy and cluttered and DSR generated class of drugs rather than the generic name of the medicine	The printout was reformatted to make it visually appealing, clear, and less cluttered. It provides information about the patient’s clinical parameters over the previous five visits, current medication, previously prescribed medication, recommended medications in their generic names, and a reminder for lifestyle advice (Figure 3).
**Knowledge and skills of nurses**	
Inability to use smart-phone/tabs	Incorporated tablet handling, typing practice, and troubleshooting during the training
Comorbid conditions assessment and management	Training on comorbid conditions, including detection using PHQ9 and AUDIT questionnaires and providing low-intensity behavioral interventions, through role play and enhanced onsite support
**Health-system barriers**	
Frequent changes in physician’s roster	Develop an onsite training and orientation program to cover all physicians in each CHC. Involve pharmacists and other supporting staff for proper implementation of the mWellcare workflow.
Authorities reluctant to depute physicians for training owing to staff shortage	
Resistance to implementing mWellcare recommended workflow	
Drugs not procured according to guidelines and unavailability of glucometer strips	Mobilize assurance from higher health officials of state/districts to ensure drug and glucometer strips availability
NCD nurse engaged in non-NCD work

**Figure 3.**
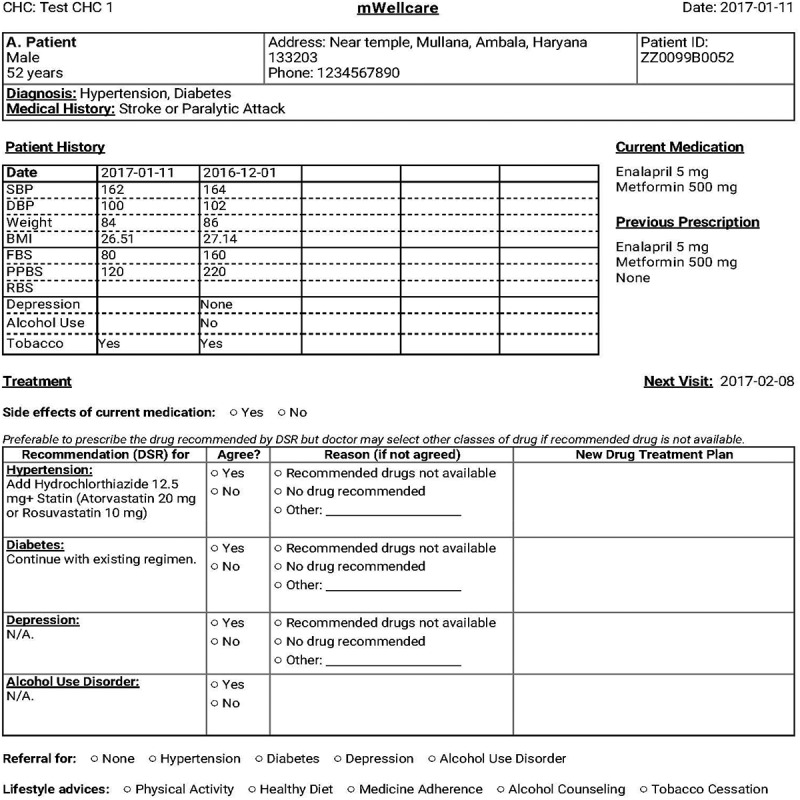
mWellcare decision-support output.

The major lessons from the pilot testing were that: (1) for smooth implementation of mWellcare, the intervention needs to be combined with workflow changes at the CHCs; (2) there needs to be refresher training of the nurses and other healthcare providers in the CHCs; (3) features on the application were simplified to increase acceptability by nurses and doctor.

## Discussion

This paper describes the systematic development of an mHealth intervention (mWellcare) aimed at integrating the management of diabetes and hypertension in primary care. Through our experience from our earlier mhealth projects [16–19], health facility assessments, and literature review, it was concluded that a lack of systematic assessment and evidence-based long-term management are the major reasons for suboptimal treatment of hypertension and diabetes in primary care. The mWellcare intervention was intended to address these gaps and was designed to optimize its utility, acceptability, and feasibility. The intervention was further refined on the basis of extensive pilot testing with the target users.

The final version of mWellcare application resulting from this development process will be used by primary care nurses and doctors, and has the following capabilities: to store and integrate longitudinal health records electronically; to provide automated guideline-recommended treatment plan, prompt referral to specialist; to generate lifestyle intervention tailored to patient clinical profile; to allow longitudinal patient monitoring with alerts to the need for changes in management; to send out automatic SMS reminders and alerts to patients; to serve as a data-collection tool for remote QA of NCD programs; and to export standards-compliant healthcare data to relevant receiving healthcare systems.

The mWellcare CDSS for hypertension and diabetes can successfully handle over 1000 permutations of clinical profiles to generate personalized recommendations through the course of the patient’s illness, for example revising drug recommendations if control is poor and recommending treatments for comorbid conditions. It also identifies permutations outside its own scope and recommends consulting with a specialist in such scenarios. Importantly, these recommendations are also guided by CVD risk profile assessments using the Framingham-Risk Equation, which has been recalibrated for the Indian population [35]. The intervention is delivered both on the mobile application and web interface, with the latter being used to store longitudinal patient data and provide remote monitoring of patient and system level implementation. The web interface also enables the clinical management algorithms to be updated in the event of new research findings on treatment effectiveness. Once these changes have been confirmed and deployed, the mWellcare mobile applications are able to automatically download the updates remotely. The mWellcare mobile application, in addition to prompts for patient inputs and the resultant DSR, also provides a graphical display of a patient’s longitudinal clinical parameters. Data about patients can be shared between physicians and nurses either electronically using android mobile devices or through the system-customized printout. The mWellcare system also generates personalized lifestyle modification advice and also provides direct-to-patient personalized behavior change messaging. The mWellcare system platform is built with Python using the Django web framework, the mobile application is driven by an XML application configuration layer with JavaRosa at the core, and the database is CouchDB. The mWellcare mobile application is an android application based on CommCare platform, which can be downloaded through the Google play store. It can work in the offline mode, but Internet connectivity is required to sync the data with the central server.

With regard to the scalability of the platform, the system is in compliance with Electronic Health Record Standards 2016, for Healthcare Information Technology notified by Ministry of Health and Family Welfare (MOHFW) [36]. Standards used in mWellcare are: ICD 10 (International Statistical Classification of Diseases and Related Health Problems 10th Revision) for diagnosis, LOINC (Logical Observation Identifiers Names and Codes) for lab results, UIDAI (Unique Identity Authority of India) Aadhaar for patient identification, HL7 (Health Level-7) for messaging and Continuity of Care Document for clinical document exchange for interoperability. Thus, mWellcare is enabled to be interoperable and send data to other standards-compliant systems like a Diabetic Registry or Integrated Health Information Platform. The application also has a number of security features to ensure confidentiality. In the mobile application, password-protected access is enabled to restrict unauthorized access. Only encrypted data are stored on the tablet. Data no longer required on the mobile application are purged from the device after submission to the server. Only the subset of data required for future visits is retained. On the server, all data are stored encrypted at rest using Advanced Encryption Standard (AES) 256-bit Symmetric Encryption. The server is Health Insurance Portability and Accountability compliant, and the infrastructure is hosted in a secure ISO 27,001 facility with biometric security. Data are backed up every 24 h, and, in the event of a catastrophic failure, the maximum period over which data would be lost owing to a corruption issue on a server is 24 h.

The mWellcare intervention has several limitations. The mHealth application developed for mWellcare intervention is a postdiagnosis decision-support tool and can only be used once the person is diagnosed with hypertension or diabetes by the physician. However, the current version of the mWellcare application can be expanded to include diagnostic functionality and to integrate with external diagnostic devices. Keeping in mind the maintenance of the software and the management of the data, the current application only permits central customization, which may limit site-specific variations. As the mWellcare application is based on the CommCare platform, it is not as perfectly tailored to specific needs of the target users as customized software could be; as one example, the layout of fields on a single screen and visualization options were limited owing to the rigid structure of the templates on the platform and predefined options for in-built reports. The benefits of customized software, for example, that users can dictate exactly how they want their software to behave and what reports should be implemented, was weighed against the potential scalability and interoperability of such customized software. Finally, the ability of mWellcare to fully achieve its intended goals will always be constrained by health system factors, such as availability of drugs and doctors at CHCs.

This is, to best of our knowledge, the first study describing the development of an mHealth intervention for integrated management of chronic conditions in a low-resource setting. The resulting mWellcare intervention aims to provide integrated management of hypertension and diabetes at the primary care level using evidence-based management protocols. The cost-effectiveness of the intervention is currently being evaluated in a cluster randomized controlled trial in India (trial registration number NCT02480062).

## Conclusion

Although the use of mHealth interventions is increasing, the literature on their development and their implementation is relatively scanty. This article describes the major gaps in the chronic disease management at public primary care setting, how these gaps can be addressed through the mHealth intervention, steps for the development, and implementation of the intervention and evaluation of the potential mhealth solution through pilot testing.

## Paper context

mWellcare is a unique mHealth intervention for integrated management of hypertension and diabetes along with comorbid conditions like depression at primary care level in public health setting in India. Although the use of mHealth interventions is increasing, the literature on their development and their implementation is relatively scanty. Publication of this research will be helpful for the future development and implementation of the mHealth intervention in low-resource settings.

## References

[1] Wang H, Naghavi M, Allen C, et al Global, regional, and national life expectancy, all-cause mortality, and cause-specific mortality for 249 causes of death, 1980–2015: a systematic analysis for the global burden of disease study 2015. Lancet. 2016;388:1459–10. 27733281 10.1016/S0140-6736(16)31012-1 PMC5388903

[2] World Health Organization Global status report on noncommunicable diseases 2014. Geneva: WHO; 2014. 10.1161/STROKEAHA.115.008097 25873596

[3] International Diabetes Federation Diabetes In India- 2015 [Internet]. Int. Diabetes Fed. 2016 [cited 2018 724]. Available from: http://www.idf.org/membership/sea/india.

[4] Thankappan KR, Sivasankaran S, Sarma PS, et al Prevalence-correlates-awareness-treatment and control of hypertension in kumarakom, kerala: baseline results of a community-based intervention program. Indian Heart J. 2006;58:28–33. 18984927

[5] Venkataraman K, Kannan AT, Mohan V. Challenges in diabetes management with particular reference to India. Int J Diabetes Dev Ctries. 2009;29:103–109. 20165646 10.4103/0973-3930.54286 PMC2822213

[6] Jonas JB, Nangia V, Matin A, et al Prevalence, awareness, control, and associations of arterial hypertension in a rural central India population: the Central India eye and medical study. Am J Hypertens. 2010;23:347–350. 20094037 10.1038/ajh.2009.276

[7] Joshi R, Chow CK, Raju PK, et al Fatal and nonfatal cardiovascular disease and the use of therapies for secondary prevention in a rural region of India. Circulation. 2009;119:1950–1955. 19332466 10.1161/CIRCULATIONAHA.108.819201

[8] Garg AX, Adhikari NKJ, McDonald H, et al Effects of computerized clinical decision support systems on practitioner performance and patient outcomes: a systematic review. JAMA. 2005;293:1223–1238. 15755945 10.1001/jama.293.10.1223

[9] Berntson J, Stewart KR, Vrany E, et al Depressive symptoms and self-reported adherence to medical recommendations to prevent cardiovascular disease: NHANES 2005-2010. Soc Sci Med. 2015;138:74–81. 26056936 10.1016/j.socscimed.2015.05.041

[10] Arshad AR, Alvi KY Frequency of depression in type 2 diabetes mellitus and an analysis of predictive factors. J Pak Med Assoc. 2016;66:425–429. 27122269

[11] Timko C, Kong C, Vittorio L, et al Screening and brief intervention for unhealthy substance use in patients with chronic medical conditions: a systematic review. J Clin Nurs. 2016;25:3131–3143. 27140392 10.1111/jocn.13244 PMC6430571

[12] Anchala R, Pinto MP, Shroufi A, et al The role of decision support system (DSS) in prevention of cardiovascular disease: a systematic review and meta-analysis. Gong Y, editor Plos ONE. 2012;7:e47064. 23071713 10.1371/journal.pone.0047064 PMC3468543

[13] Ministry of Health and Family Welfare Indian public health standards [Internet]. 2012 [cited 2018 724]. Available from: http://www.nhm.gov.in/nhm/nrhm/guidelines/indian-public-health-standards.html.

[14] Ministry of Health & Family Welfare National programme for prevention and control of cancer, diabetes, cardiovascular diseases and stroke (NPCDCS): operation guidelines. New Delhi, India: Ministry of Health and Family Welfare, Government of India; 2010.

[15] Craig P, Dieppe P, Macintyre S, et al Developing and evaluating complex interventions: the new medical research council guidance. BMJ. 2008;337:a1655. 18824488 10.1136/bmj.a1655 PMC2769032

[16] Ajay VS, Jindal D, Roy A, et al Development of a smartphone-enabled hypertension and diabetes mellitus management package to facilitate evidence-based care delivery in primary healthcare facilities in India: the mPower heart project. J Am Heart Assoc. 2016;5:e004343. 28003248 10.1161/JAHA.116.004343 PMC5210443

[17] Tian M, Ajay VS, Dunzhu D, et al A cluster-randomized, controlled trial of a simplified multifaceted management program for individuals at high cardiovascular risk (simcard trial) in rural Tibet, China, and Haryana, India. Circulation. 2015;132:815–824. 26187183 10.1161/CIRCULATIONAHA.115.015373 PMC4558306

[18] Anchala R, Di Angelantonio E, Prabhakaran D, et al Development and validation of a clinical and computerised decision support system for management of hypertension (DSS-HTN) at a primary health care (PHC) setting. Plos ONE. 2013;8:e79638. 24223984 10.1371/journal.pone.0079638 PMC3818237

[19] Ali MK, Singh K, Kondal D, et al Effectiveness of a multicomponent quality improvement strategy to improve achievement of diabetes care goals: a randomized, controlled trial. Ann Intern Med. 2016;165:399–408. 27398874 10.7326/M15-2807 PMC6561084

[20] James PA, Oparil S, Carter BL, et al 2014 evidence-based guideline for the management of high blood pressure in adults. JAMA. 2014;311:507. 24352797 10.1001/jama.2013.284427

[21] Members AF, Mancia G, Fagard R, et al 2013 ESH/ESC guidelines for the management of arterial hypertension. Eur Heart J. 2013;34:2159–2219. 23771844 10.1093/eurheartj/eht151

[22] World Health Organization Prevention of cardiovascular disease guidelines for assessment and management of cardiovascular risk. Geneva: World Health Organization; 2007.

[23] National Institute for Health and Care Excellence (NICE) Hypertension in adults: diagnosis and management [Internet]. National Institute for Health and Care Excellence; 2011 Available from: https://www.nice.org.uk/guidance/cg127/resources/hypertension-in-adults-diagnosis-and-management-35109454941637.

[24] Ministry of Health and Family Welfare National Programme for Prevention and Control of Cancer, Diabetes, Cardiovascular Disease and Stroke (NPCDCS): A Manual for Medical Officer. New Delhi, India: Ministry of health and family welfare, Government of India; 2008.

[25] World Health Organization mhGAP Intervention Guide for mental, neurological and substance use disorders in non-specialized health settings. Geneva: World Health Organization; 2010. 23741783

[26] CommCare | Dimagi [Internet] CommCare. [cited 2018 7 24]. Available from: http://www.dimagi.com/products/.

[27] Flaming A, Canty M, Javetski G, et al The commcare evidence base for frontline workers [Internet]. 2015 [cited 2018 724]. Available from: https://healthmarketinnovations.org/sites/default/files/CommCare_Impact.Evaluation._0.pdf.

[28] Crosson JC, Heisler M, Subramanian U, et al Physicians’ perceptions of barriers to cardiovascular disease risk factor control among patients with diabetes: results from the translating research into action for diabetes (TRIAD) study. J Am Board Fam Med. 2010;23:171–178. 20207927 10.3122/jabfm.2010.02.090125

[29] Düsing R Overcoming barriers to effective blood pressure control in patients with hypertension. Curr Med Res Opin. 2006;22:1545–1553. 16870079 10.1185/030079906X120995

[30] Menon VU, Guruprasad U, Sundaram KR, et al Glycaemic status and prevalence of comorbid conditions among people with diabetes in Kerala. Natl Med J India. 2008;21:112–115. 19004140

[31] Pakhare A, Kumar S, Goyal S, et al Assessment of primary care facilities for cardiovascular disease preparedness in Madhya Pradesh, India. BMC Health Serv Res. 2015;15:408. 26399634 10.1186/s12913-015-1075-x PMC4580030

[32] Mendis S, Al Bashir I, Dissanayake L, et al Gaps in capacity in primary care in low-resource settings for implementation of essential noncommunicable disease interventions. Int J Hypertens. 2012;2012:584041. 23251789 10.1155/2012/584041 PMC3517842

[33] Kroenke K, Spitzer RL, Williams JBW The PHQ-9. J Gen Intern Med. 2001;16:606–613. 11556941 10.1046/j.1525-1497.2001.016009606.x PMC1495268

[34] Babor TF, Higgins-Biddle Ohn C, Saunders JB, et al AUDIT, The alcohol use disorders identification test. Geneva: World Health Organization; 2001.

[35] D’Agostino RB, Vasan RS, Pencina MJ, et al General cardiovascular risk profile for use in primary care: the Framingham heart study. Circulation. 2008;117:743–753. 18212285 10.1161/CIRCULATIONAHA.107.699579

[36] Ministry of Health and Family Welfare Electronic health record (EHR) standards for India. New Delhi, India: Ministry of Health and Family Welfare, Government of India; 2016.

